# Olfactory and Visuospatial Learning and Memory Performance in Two Strains of Alzheimer's Disease Model Mice—A Longitudinal Study

**DOI:** 10.1371/journal.pone.0019567

**Published:** 2011-05-05

**Authors:** Matthew Phillips, Erik Boman, Hanna Österman, David Willhite, Matthias Laska

**Affiliations:** 1 Department of Neurobiology, Yale University School of Medicine, New Haven, Connecticut, United States of America; 2 Department of Physics, Yale University, New Haven, Connecticut, United States of America; 3 Department of Physics, Chemistry and Biology, Section of Zoology, Linköping University, Linköping, Sweden; Duke University, United States of America

## Abstract

Using a longitudinal study design, two strains of Alzheimer's disease (AD) model mice, one expressing β-amyloid plaques and one expressing Tau protein-associated neurofibrillary tangles were assessed for olfactory and visuospatial learning and memory and their performance compared to that of age-matched controls. No significant difference between AD and control mice was found in the initial set of olfactory tasks performed at 6 months of age whereas both strains of AD mice performed significantly poorer than the controls in visuospatial learning at this age. Subsequent tests performed on the same individual animals at 7, 8, 9, 11, 13, 15, and 18 months of age also failed to find systematic differences in olfactory performance between AD and control mice. In contrast, the AD mice performed consistently poorer than the controls in visuospatial re-learning tests performed at these ages. With most olfactory tasks, both AD and control mice displayed a marked decrease in performance between testing at 15 and 18 months of age. These results show that the two strains of AD model mice do not display an olfactory impairment in a time course consistent with human AD, but are impaired in visuospatial capabilities. The marked age-related changes observed with the olfactory tasks in both AD and control mice suggest that the observed lack of an AD-related olfactory impairment is not due to an insensitivity of the tests employed. Rather, they suggest that the olfactory system of the two AD mouse model strains may be surprisingly robust against AD-typical neuropathologies.

## Introduction

Alzheimer's disease (AD) is a neurodegenerative disorder characterized by progressive cognitive and sensory impairment. It is the most common form of dementia and affects about 10% of the human population at the age of 65, rising to about 50% at the age of 85 [1]. Although the causes underlying AD are not fully understood, it is commonly agreed that the accumulation of β-amyloid plaques and Tau protein-associated neurofibrillary tangles in the brain are characteristic for the neuropathology of the disease.

Recent progress in the engineering of transgenic mice now allows us to study animals that develop the same type of neuropathologies as humans suffering from AD [2]. However, surprisingly little is known about the sensory and cognitive changes that may result from the progressing neuropathologies, raising the question as to the validity and usefulness of such animal models [3]. Most studies on AD-related impairments in sensory or cognitive performance using AD model mice adopted a cross-sectional design, where animals are tested only once at a given time during their life span and are then sacrificed to assess their neuropathology. The disadvantages of this study design are that it requires a comparatively high number of animals, that measures of long-term re-learning are rather restricted, and the progression of AD- or age-related changes in all measures of performance can only be inferred indirectly - across (rather than within) animals. We therefore consider it useful to complement this approach with studies adopting a longitudinal design, that is, animals are tested repeatedly at several times during their lifespan. The longitudinal study design has the advantage that the onset and progression of AD- or age-related changes in all measures of sensory and cognitive performance can be measured directly, within (rather than across) individual animals. Further, it has the advantage that only a comparatively small number of animals is required and measures of long-term re-learning are not as limited as in the cross-sectional study design. The disadvantage of the longitudinal study design is that correlations between the onset and severity of neuropathologies and the onset and severity of sensory and cognitive changes can be inferred only indirectly, by using neuropathological data from other animals of the same strain. However, the two AD model mouse strains used in the present study have been characterized thoroughly with regard to the onset, progression and severity of their neuropathologies [4]–[7] and thus correlations with the sensory and cognitive performance of longitudinally tested animals should be reliable.

It was therefore the aim of the present pilot study to assess the onset, progression and severity of sensory and cognitive changes in two strains of AD model mice, adopting a longitudinal study design. Given the strong reliance of mice on their sense of smell in virtually all behavioral contexts, and that an impaired sense of smell is one of the earliest clinical symptoms of human AD [8]–[10], we decided to focus on this sensory modality with regard to characterizing AD-related sensory and cognitive changes. However, to assess whether such changes are modality-specific we also included a test of visuospatial and thus non-olfactory learning and memory into our battery of tests. Using one mouse strain that expresses β-amyloid plaques and one mouse strain that expresses Tau protein-associated neurofibrillary tangles allowed us to distinguish between the impact of these two AD-related neuropathologies on sensory and cognitive performance. Testing a strain of healthy control mice without AD-typical neuropathologies in parallel allowed us to distinguish between AD-related and normal aging-related changes in sensory and cognitive performance.

## Materials and Methods

### Ethics Statement

The experiments reported here comply with the *Guide for the Care and Use of Laboratory Animals* (National Institutes of Health Publication no. 86-23, revised 1985) and were performed according to a protocol approved by the Yale University Institutional Animal Care and Use Committee (protocol #11100).

### Animals

Testing was carried out using male adult mice of three different strains:

Swede mice: Three mice of the strain B6.Cg-Tg(APPswe,PSEN1dE9)85Dbo/J were used. This strain expresses a chimeric mouse/human amyloid precursor protein (Mo/HuAPP695swe) and a mutant human presenilin 1 (PS1-dE9). These animals, which will be referred to as Swede mice for the Swedish origin of the mutation, display β-amyloid plaques and spatial learning deficits at six months of age [5].

Tau mice: three mice of the strain B6.Cg-MAPT^tm1(EGFP)Klt^Tg(MAPT)8cPdav/J were used. This strain expresses microtubule-associated protein Tau (MAPT). Hyperphosphorylated Tau protein accumulates in neuronal cell bodies and dendrites as early as three months of age [4].

Control mice: three mice of the strain C57BL/6J were used. This strain has a genetic background similar to that of the Swede and Tau mice but lacks the mutations causing AD-typical neuropathologies.

All mice were obtained from Jackson Laboratories (Bar Harbor, ME). They were six months old at the beginning of the study. Maintenance of the mice has been described in detail elsewhere [11]. During the experiments, the animals were kept on a water deprivation schedule of 1.5 ml of water per day.

### Stimuli

A set of 21 odorants was used (Table 1). With all discrimination tasks the odorants were presented at a gas phase concentration of 1 ppm (parts per million) and with all sensitivity tasks testing started at this concentration and then proceeded with lower concentrations at ten-fold steps (0.1 ppm, and 0.01 ppm). Gas phase concentrations for all odorants were calculated using published vapor pressure values and formulae provided by [12]. All substances were obtained from Sigma-Aldrich (St. Louis, MO) and had a nominal purity of at least 99%. They were diluted using near-odorless diethyl phthalate (CAS# 84-66-2) as the solvent. The rationale for using this set of odorants was that they have been successfully used in previous studies with mice and are thus known to be detectable and discriminable at the concentrations used here [11], [13]–[17].

**Table 1:** Odorants used.

no.	odorant	CAS#	Liquid dilution*
1	n-pentyl acetate	628-63-7	1∶220
2	eugenol	97-53-0	1∶5
3	anethole	104-46-1	1∶10
4	1,8-cineole	470-82-6	1∶85
5	S-(+)-2-butanol	4221-99-2	1∶1,400
6	R-(–)-2-butanol	14898-79-4	1∶1,400
7	n-hexanal	66-25-1	1∶420
8	S-(+)-carvone	2244-16-8	1∶12
9	R-(–)-carvone	6485-40-1	1∶12
10	(+)-limonene	5989-27-5	1∶79
11	(–)-limonene	5989-54-8	1∶79
12	(+)-isopulegol	104870-56-6	1∶23
13	(–)-isopulegol	89-79-2	1∶23
14	(+)-rose oxide	16409-43-1	1∶12
15	(–)-rose oxide	876-17-5	1∶12
16	(+)-perilla aldehyde	5503-12-8	1∶9
17	(–)-perilla aldehyde	18031-40-8	1∶9
18	(+)-limonene oxide	1195-92-2	1∶23
19	(–)-limonene oxide	203719-54-4	1∶23
20	(+)-fenchone	4695-62-9	1∶44
21	(–)-fenchone	7787-20-4	1∶44

*Note that the headspace above all liquid dilutions was further air-diluted by a factor of 40 within the olfactometer to provide the desired gas phase concentration at the odor port.

### Olfactory discrimination and sensitivity tests

Olfactory performance of the mice was assessed using an automated liquid-dilution olfactometer (Knosys, Tampa, FL). Mice were trained using standard operant conditioning procedures [18] to insert their snout into the odor sampling port of a test chamber. This triggered the 2 s presentation of either an odorant used as the rewarded stimulus (S+) or an alternative odorant used as the unrewarded stimulus (S–). Licking at a steel tube providing 2.5 µl of water reinforcement in response to presentation of the S+ served as the operant response.

Olfactory discrimination performance was assessed by testing the animals' ability to distinguish between a given odorant used as the S+, and an alternative odorant used as the S–. Five blocks of 20 trials (totaling 50 S+ and 50 S– trials in pseudorandomized order) using a given stimulus pair were conducted per animal and task.

Olfactory sensitivity was assessed by testing the animals' ability to discriminate between increasing dilutions of n-hexanal as S+ and a blank stimulus (headspace of the solvent) as S–. One block of 20 trials of n-hexanal presented at a gas phase concentration of 1 ppm was followed by 5 blocks of 20 trials each of the same odorant presented at 0.1 ppm and 0.01 ppm, respectively.

### Visuospatial learning and memory test

A visuospatial learning test was performed to assess the animals' ability for non-olfactory learning and memory (as assessed by re-learning). The test was performed using a T-shaped cardboard divider that was inserted into an animal's home cage creating two equally sized compartments one of which had a black wall and one had a white wall. Both walls were sealed with transparent tape and cleaned periodically with 90% ethanol, and allowed to dry, to ensure that the animals used only visual and not olfactory cues. The two compartments took up a third of the total area of the cage. Before each test trial, another piece of cardboard was placed in front of the compartments like a curtain, sealing the compartments off from the rest of the cage, which was designated as the "starting area". The animal was placed in the starting area and the curtain was raised revealing the two compartments. If the animal then entered the compartment that beforehand had been assigned as the rewarded compartment, it was rewarded with 0.1 ml of water given from a syringe lowered into the cage directly in front of the colored wall. The mouse was then placed back in the starting area with the curtain sealing off the compartments and the test was repeated. If the animal entered the unrewarded compartment, it was placed back into the starting area without getting a water reward. The test was repeated 10 times per day for 7 consecutive days, occurring at the same time and under identical lighting conditions. The number of correct decisions for each mouse per day was recorded. After acquiring the task, when tested in total darkness, all animals failed to engage in the task, and make obvious behavioral choices. Two of the animals in each group of mice were always rewarded for entering the white compartment and one of them was always rewarded for entering the black compartment.

### Olfactory habituation/dishabituation test

The animals' olfactory abilities at the age of 18 months were further assessed using an olfactory habituation/dishabituation test. The animals were presented with an odorant A for two minutes, followed by a two-minute interval without odorant. After four such two-minute presentations of odorant A and another two-minute interval without odorant, a novel odorant B was presented for two minutes. During all odorant presentations the time that an animal spent investigating the odorant was recorded. Investigation was defined as the animal sniffing while orienting itself towards the odorized object with its snout within two cm from the object. The odorized object was a filter paper (24 mm diameter) soaked with µl of odorant in an upside-down petridish (35 mm diameter) placed on top of the top mesh of the animal's home cage. This prevented the animals from touching the odorized object but allowed them to smell the odorant. The same type of object, soaked with 200 µl of water was presented four times for two minutes each with two-minute intervals on the day prior to the habituation/dishabituation test. This was done in order to minimize the possibility that interest in the object was due to the novelty of the object per se instead of the odorant. Two of the animals in each group of mice were presented with vanilla extract (Stop & Shop, Boston, MA, diluted 1∶2 in demineralized water) as odorant A and vermouth extract (Martini & Rossi Vermouth, Miami, FL, undiluted) as odorant B, and the the third animal of a given group was presented with the reversed order of odorants.

### Experimental design

#### Initial olfactory tasks

In order to train the animals to operate the olfactometer, a series of task acquisition steps were performed. These included shaping in which the animals were presented with only n-pentyl acetate as S+ and were rewarded for every poking their head into the odor port and licking at the water spout. After acquiring this operant response the animals were then trained to respond to different S+ and S– combinations in a series of initial olfactory tasks for two sessions of 100 trials each (see Table 2 for a summary of stimulus combinations).

**Table 2:** Stimulus combinations used during initial task acquisition.

Olfactory task (task number)	Stimulus combination
Initial stimulus pair (1)	1 vs 2
First negative transfer (2)	1 vs 3
First positive transfer (3)	4 vs 3
Second negative transfer (4)	4 vs 2
First double transfer (5)	5 vs 6
Second positive transfer (6)	7 vs 2
Third negative transfer (7)	7 vs B

Numbers of stimuli as in Table 1. B: blank stimulus (headspace of solvent).

#### Longitudinal olfactory testing

After completion of the series of initial olfactory tasks the mice were tested on a series of olfactory tasks at the age of 7, 8, 9, 11, 13, 15, and 18 months of age, respectively (Table 3). This was done to determine the onset and progression of possible olfactory impairment in the two strains of AD model mice. Control mice were tested in parallel to distinguish possible age-related changes in olfactory performance from AD-related changes. Task 1 assessed changes in long-term odor re-learning as the same stimulus pair had been presented to the animals during initial task acquisition and during each of the subsequent tests. Task 2 assessed changes in the ability to acquire a new double transfer task. Task 3 again assessed changes in long-term odor re-learning as the same stimulus pair had been presented to the animals for the first time on the previous test, one, two or three months previously. Task 4 assessed changes in the ability to learn a stimulus reversal. To this end, the animals were presented with a pair of stimuli that they had been presented with on the previous test, one, two or three months previously, they were trained to criterion and then presented with the same stimuli as on the day before but now with reversed reward values (that is: the former S+ was now the S–, and vice versa). Task 5 assessed changes in the ability to detect stimulus concentrations lower than 1 ppm by presenting a familiar stimulus as S+ consecutively at 1 ppm, 0.1 ppm, and 0.01 ppm against a blank stimulus as S– (see Table 2 for a summary of stimulus combinations used during the different testing periods). Tasks 1–4 are commonly regarded to differ markedly in their degree of difficulty allowing us to assess possible correlations between progressing AD neuropathologies and/or age on the one hand and task-solving capabilities on the other hand in a graded manner. After completion of the olfactory tasks one week of visuospatial learning/memory (re-learning) testing was performed. It took about 3 weeks in total to complete one round of olfactory and visuospatial tests so that about one week without testing took place until the next round of testing started.

**Table 3:** Stimulus combinations used during subsequent tests.

	Age at testing in months						
Olfactory task	7	8	9	11	13	15	18
LT re-learning 1	1 vs 2	1 vs 2	1 vs 2	1 vs 2	1 vs 2	1 vs 2	1 vs 2
Double transfer	8 vs 9	10 vs 11	12 vs 13	14 vs 15	16 vs 17	18 vs 19	20 vs 21
LT re-learning 2	5 vs 6	8 vs 9	10 vs 11	12 vs 13	14 vs 15	16 vs 17	18 vs 19
Reversal task	6 vs 5	9 vs 8	11 vs 10	13 vs 12	15 vs 14	17 vs 16	19 vs 18
Sensitivity test	7 vs B	7 vs B	7 vs B	7 vs B	7 vs B	7 vs B	7 vs B

Numbers of stimuli as in Table 1. B: blank stimulus (headspace of solvent) LT: long-term.

### Data analysis

#### Olfactory tasks

For each individual animal, the percentage of correct choices from 100 decisions per stimulus pair was calculated. Correct choices consisted both of licking in response to presentation of the S+ and not licking in response to the S–, and errors consisted of animals showing the reverse pattern of operant responses. Additionally, the percentage of correct choices in the first block of 20 trials per task (comprising 10 S+ and 10 S– trials in pseudorandomized order) was analyzed.

#### Visuospatial tasks

For each individual animal, the percentage of correct choices from 10 decisions per day was calculated. Correct choices consisted of entering the compartment assigned as S+, and errors consisted of entering the compartment assigned as S–.

With both kinds of tasks, significance levels were determined by calculating binomial z-scores corrected for continuity from the number of correct and false responses for each individual and condition. Comparisons of performance between groups of animals were made using the Mann-Whitney U-test for independent samples, and comparisons of performance within groups of animals were made using the Wilcoxon signed-rank test for related samples. If not otherwise mentioned, the alpha level was set at 0.01.

### Histology

After completing the behavioral tests the mice were transcardially perfused with phosphate-buffered saline (PBS), then 4% paraformaldehyde (pH 6.5) then sectioned coronally at 50 µm. Sections were stained in 0.1% Thioflavin T in 50% ethanol for 10 min at room temperature, washed three times with 80% ethanol, then washed three times with water and then returned to PBS. Sections were then counterstained in 0.3% Sudan Black B in 70% ethanol for 5 min, washed three times with 70% ethanol, then two times with PBS [19]. Mice were also assayed for endogenous GFP protein expression. GFP labeling (in the tau mouse, as a result of a GFP sequence upstream of the endogenous tau promoter) indicated hyperphosphorylated MAPT overexpression accumulating as aggregated paired helical filaments in the soma and dendrites of neurons, and confirms the presence of neurofibrillary tangles as previously demonstrated. [4], [20].

## Results

### Initial olfactory tasks

**Figure 1:**
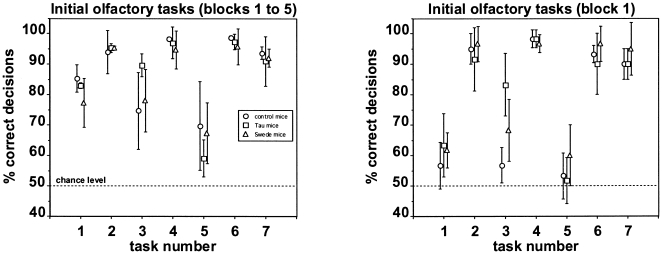
Performance in the seven initial olfactory tasks performed at six months of age. Each data point [control mice (circles), Tau mice (squares), and Swede mice (triangles)] represents the percentage (mean ± SD) of correct decisions per task across the first five blocks of 20 trials performed per animal and task (left panel), and in the first block of 20 trials (right panel). Task numbers as in Table 2. The dotted line indicates the chance level of performance.

Figure 1 summarizes the performance of the three mouse strains in the initial set of olfactory tasks. The mean percentage of correct decisions across the first five blocks of 20 trials performed per animal and task (Fig. 1, left panel) shows that all three mouse strains performed significantly above chance in all tasks (binomial test, p<0.01). Interindividual variability with a given task was less than 10% in the majority of cases (see SDs). Task 5, the first double transfer task where the animal must associate reward with a new set of odorants, was significantly more difficult for all three mouse strains compared to tasks 2, 4, 6, and 7, which represent positive and negative transfer tasks, where only the rewarded or unrewarded odorants are changed respectively (Wilcoxon, p<0.05). The across-task patterns of performance correlated significantly between the three mouse strains (Spearman, 0.93≤ r_s_ ≤0.96, p<0.01) indicating that tasks that were relatively difficult for one of the mouse strains were also relatively difficult for the other two mouse strains. Accordingly, there were no systematic differences in performance between control mice, Tau mice, and Swede mice in the initial olfactory tasks performed at six months of age.

Exactly the same pattern of performance can be seen when considering only the percentage of correct decisions in the first block of 20 trials performed per animal and task (Figure 1, right panel). The difference between mean overall performance (Figure 1, left panel) and mean block 1 performance (Figure 1, right panel) was usually less than 10%, indicating a fast learning process with most tasks. The only exceptions to this were task 1, the very first odorant pair to be discriminated, and task 5, the first double transfer task: here, the difference between block 1 performance and overall performance ranged from 15.6% (Swede mice) to 28.6% (control mice). Accordingly, in the very first task, the animals reached criterion in block 3 (control mice and Tau mice) and block 4 (Swede mice), respectively, whereas with the exception of task 5 (the double-transfer task) they usually reached criterion in blocks 1 or 2 with the other tasks (data not shown). Similar to overall performance, there were no systematic differences in block 1 performance, and thus in speed of olfactory learning, between control mice, Tau mice, and Swede mice in the initial olfactory tasks performed at six months of age.

With regard to the initial test of olfactory sensitivity, all individual mice of all three strains were clearly able to discriminate 1 ppm, 0.1 ppm, and 0.01 ppm of hexanal from the solvent (data not shown). Thus, there were no systematic differences between control mice, Tau mice, and Swede mice in their ability to detect these concentrations when tested at six months of age.

### Visuospatial learning test

**Figure 2:**
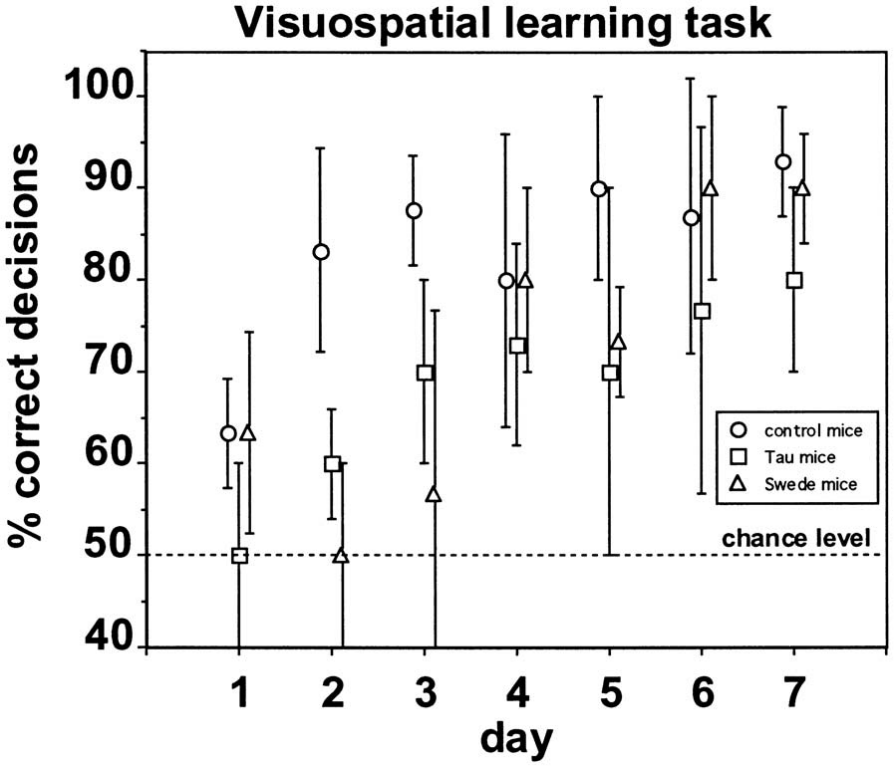
Performance in the visuospatial learning task performed at six months of age. Each data point [control mice (circles), Tau mice (squares), and Swede mice (triangles)] represents the percentage (mean ± SD) of correct decisions per animal and day. The dotted line indicates the chance level of performance.

Figure 2 shows the mean performance of the three mouse strains in the visuospatial learning test. All three mouse strains displayed a significant learning tendency across the seven days of testing (Spearman, 0.81≤ r_s_ ≤0.94, p<0.05) and reached the criterion of 70% correct decisions (corresponding to p<0.05, binomial test) on day 2 (control mice), day 3 (Tau mice), and day 4 (Swede mice), respectively. The more stringent criterion of 76.7% correct decisions (corresponding to p<0.01, binomial test) was reached on day 2 (control mice), day 6 (Tau mice), and day 4 (Swede mice), respectively. The differences in performance between the control mice on the one hand and the Tau mice and Swede mice on the other hand were significant on days 2 and 3 (Mann-Whitney, p<0.05), indicating faster visuospatial learning in the control mice compared to the two groups of AD mice when tested at six months of age.

### Longitudinal olfactory testing

**Figure 3:**
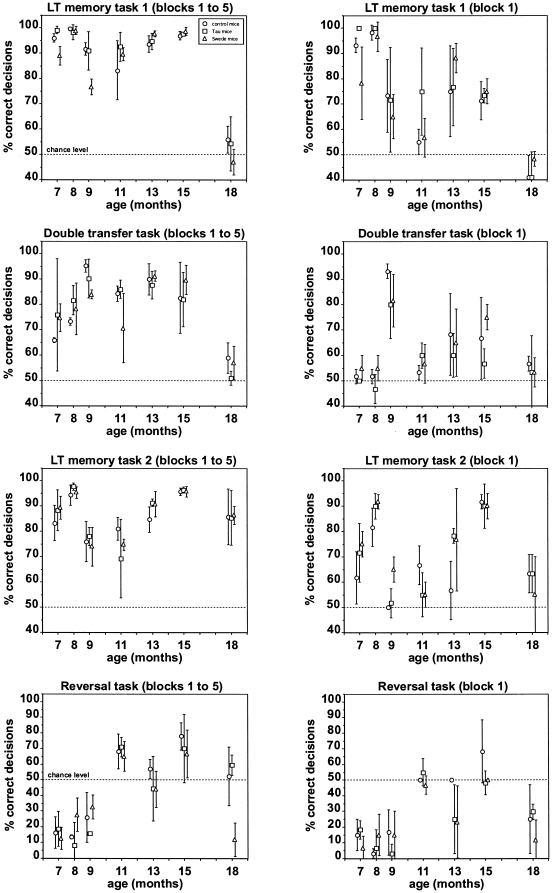
Performance in four different olfactory tasks performed at 7, 8, 9, 11, 13, 15, and 18 months of age. Each data point [control mice (circles), Tau mice (squares), and Swede mice (triangles)] represents the percentage (mean ± SD) of correct decisions across the five blocks of 20 trials performed per animal and task (left panel), and in the first block of 20 trials (right panel). The dotted line indicates the chance level of performance.

Figure 3 summarizes the mean performance of the three mouse strains in the olfactory tests performed at 7, 8, 9, 11, 13, 15, and 18 months of age.

#### Long-term re-learning task 1

In this task, all three mouse strains performed significantly above chance in all tests except for the last one performed at 18 months of age. Interindividual variability in mean overall performance (left panel) was generally low (see SDs). There were no systematic changes in mean overall performance (left panel) between testing at 7 months of age and 15 months of age (Spearman, −0.14≤ r_s_ ≤0.31, p>0.05), followed by a marked drop in performance to chance level at 18 months of age with all three mouse strains. The difference between mean overall performance (left panel) and mean block 1 performance (right panel) did not change systematically between testing at 7 months of age and 15 months of age (Spearman, 0.37≤ r_s_ ≤0.43, p>0.05) indicating a lack of systematic changes in the speed of re-learning of this task in all three mouse strains. The across-test patterns of overall performance correlated significantly between the three mouse strains (Spearman, 0.93≤ r_s_ ≤0.96, p<0.01). This is also true for block 1 performance (right panel). Accordingly, there were no systematic differences in performance between control mice, Tau mice, and Swede mice in this task.

#### Double transfer task

In this task, both the rewarded and unrewarded odorants are replaced. All three mouse strains performed significantly above chance except for the last test performed at 18 months of age. Interindividual variability in mean overall performance (left panel) was generally low (see SDs). There was a non-significant trend for an increase in mean overall performance (left panel) between testing at 7 months of age and 15 months of age (Spearman, 0.49≤ r_s_ ≤0.60, p>0.05), followed by a marked drop in performance to chance level at 18 months of age with all three mouse strains. The difference between mean overall performance (left panel) and mean block 1 performance (right panel) did not change systematically between testing at 7 months of age and 15 months of age (Spearman, −0.23≤ r_s_ ≤0.37, p>0.05) indicating a lack of systematic changes in the speed of learning of this task in all three mouse strains. The across-test patterns of overall performance correlated significantly between the three mouse strains (Spearman, 0.53≤ r_s_ ≤0.98, p<0.05). This is also true for block 1 performance (right panel). Accordingly, there were no systematic differences in performance between control mice, Tau mice, and Swede mice in this task.

#### Long-term re-learning task 2

In this task, all three mouse strains performed significantly above chance in all tests including the last one performed at 18 months of age. Interindividual variability in mean overall performance (left panel) was generally low (see SDs). There were no systematic changes in mean overall performance (left panel) between testing at 7 months of age and 18 months of age (Spearman, −0.07≤ r_s_ ≤0.39, p>0.05) in all three mouse strains. However, with all three mouse strains performance dropped between testing at 15 months of age and 18 months of age. The difference between mean overall performance (left panel) and mean block 1 performance (right panel) did not change systematically between testing at 7 months of age and 18 months of age (Spearman, −0.11≤ r_s_ ≤0.32, p>0.05) indicating a lack of systematic changes in the speed of re-learning of this task in all three mouse strains. The across-test patterns of overall performance correlated significantly between the three mouse strains (Spearman, 0.82≤ r_s_ ≤0.93, p<0.05). This is also true for block 1 performance (right panel). Accordingly, there were no systematic differences in performance between control mice, Tau mice, and Swede mice in this task.

#### Reversal task

In this task, all three mouse strains performed significantly above chance only in the tests performed at 11 and 15 months of age, whereas they failed with the same type of task performed at 7, 8, 9, 13, and18 months of age. Interindividual variability in mean overall performance (left panel) was generally low (see SDs). There was a clear trend for an increase in mean overall performance (left panel) between testing at 7 months of age and 15 months of age which was significant for the control mice and the Swede mice (Spearman, 0.89≤ r_s_ ≤0.94, p<0.05), but fell short of significance for the Tau mice (Spearman, r_s_ = 0.66, p>0.05). With all three mouse strains performance dropped between testing at 15 months of age and 18 months of age. The difference between mean overall performance (left panel) and mean block 1 performance (right panel) increased significantly between testing at 7 months of age and 15 months of age with the Tau mice and the Swede mice (Spearman, r_s_ = 0.94, p<0.05), but not in the control mice (Spearman, r_s_ = 0.47, p>0.05) indicating a decrease in the speed of learning of this task in the two AD mouse strains.

The across-test patterns of overall performance correlated significantly between the three mouse strains (Spearman, 0.75≤ r_s_ ≤0.89, p<0.05). This is also true for block 1 performance (right panel). Accordingly, there were no systematic differences in performance between control mice, Tau mice, and Swede mice in this task.

With regard to the test of olfactory sensitivity, all individual mice of all three strains were clearly able to discriminate 1 ppm, 0.1 ppm, and 0.01 ppm of hexanal from the solvent when tested at 7, 8, 9, 11, 13, and 15 months of age (data not shown). At 18 months of age, in contrast, all animals of all three strains failed with detecting the lowest concentration of hexanol (0.01 ppm), whereas they were still able to detect the higher concentrations of 1 ppm and 0.1 ppm. Thus, there were no systematic differences between control mice, Tau mice, and Swede mice in olfactory sensitivity.

### Visuospatial re-learning test

**Figure 4:**
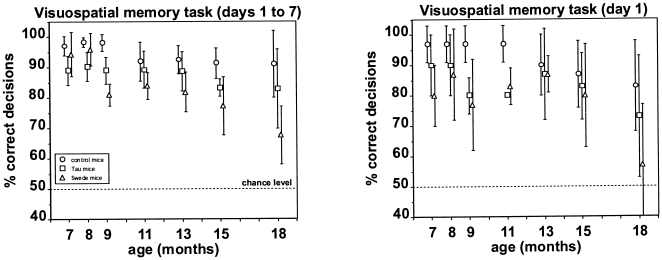
Performance in the visuospatial memory task performed at 7, 8, 9, 11, 13, 15, and 18 months of age. Each data point [control mice (circles), Tau mice (squares), and Swede mice (triangles)] represents the percentage (mean ± SD) of correct decisions across the seven days of testing (left panel), and on the first day of testing (right panel). The dotted line indicates the chance level of performance.

Figure 4 summarizes the mean performance of the three mouse strains in the visuospatial re-learning test performed at 7, 8, 9, 11, 13, 15, and 18 months of age. The mean performance across all seven test days performed at a given age (left panel) showed that all three mouse strains performed significantly above chance except for the Swede mice at 18 months of age (binomial test, p<0.05). The same is true when only considering the mean performance on the first test day performed at a given age (right panel). Interindividual variability in mean performance across all seven test days (left panel) was generally low (see SDs). There was a significant decrease in mean overall performance (left panel) between testing at 7 months of age and 18 months of age in all three mouse strains (Spearman, −0.86≤ r_s_ ≤ −0.79, p<0.05). At all seven tests between 7 months and 18 months of age, the control mice performed better than the Tau mice and the Swede mice. This is true both when considering their overall performance (left panel) and when considering only their performance on the first test day performed at a given age (right panel). This difference in overall performance (left panel) was significant at 7, 8, 9, and 15 months of age with the Tau mice, and at 9, 11, 13, 15, and 18 months of age with the Swede mice (Mann-Whitney, p<0.05). The Tau mice, in turn, performed better than the Swede mice at 9, 11, 13, 15, and18 months of age, and this difference was significant at 9 and 18 months of age (Mann-Whitney, p<0.05).

The difference between the mean performance across all seven test days performed at a given age (left panel) and mean performance only on the first day of testing at a given age (right panel) increased significantly with the Swede mice and the Tau mice (Spearman, 0.61≤ r_s_ ≤0.75, p<0.05) indicating a decrease in the speed of re-learning of this task. No such loss in re-learning speed was found with the control mice. Thus, there were systematic differences between control mice, Tau mice, and Swede mice in visuospatial re-learning.

### Olfactory habituation/dishabituation test

**Figure 5:**
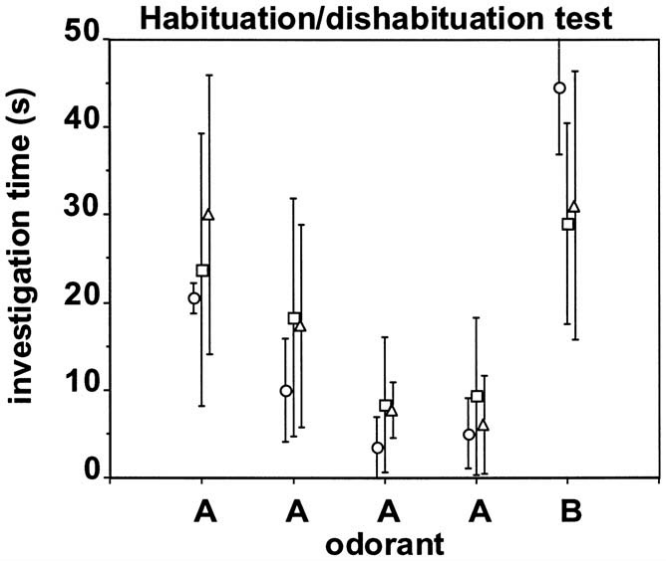
Performance in the olfactory habituation/dishabituation task performed at 18 months of age. Each data point [control mice (circles), Tau mice (squares), and Swede mice (triangles)] represents the investigation time (mean ± SD) in seconds during four consecutive 2-minute presentations of an odorant A followed by one 2-minute presentation of a novel odorant B.

Figure 5 summarizes the performance of the three mouse strains in the olfactory habituation/dishabituation test performed at 18 months of age. All three mouse strains showed a significant and at least three-fold decrease in investigation time across the four consecutive presentations of the habituation odorant A (Wilcoxon, p<0.05). Similarly, all three mouse strains showed a significant and at least three-fold increase in investigation time with the novel odorant B compared to the fourth presentation of the habituation odorant A (Wilcoxon, p<0.05). This suggests that all animals were clearly able to detect and discriminate between the two odorants. Investigation time did not differ significantly between the three mouse strains at any of the five odorant presentations. However, the increase in investigation time between the fourth presentation of odorant A and the first presentation of odorant B was higher in the control mice (factor of increase: 8.9) than in the Swede mice (factor of increase: 5.2) and the Tau mice (factor of increase: 3.1).

### Histology

After completing the behavioral tests the mice were sacrificed at 18 months of age. Histological sections confirmed the presence of β-amyloid plaques in the Swede mice. Endogenous GFP protein expression under the control of the upstream endogenous tau promoter was observed as punctuate labeling in all three Tau mice. This labeling confirms MAPT expression in the Tau mice used in this study and indicates the presence of neurofibrillary tangles as previously demonstrated [19]. These results confirmed genotyping tests (data not shown) performed prior to initiating the study. Neither β-amyloid plaques nor neurofibrillary tangles were found in the control mice.

## Discussion

The results of the present study demonstrate that the two strains of AD model mice employed here do not display an AD-related impairment in olfactory performance, but do show an impairment in visuospatial learning and memory (re-learning) compared to control mice. Further, marked age-related changes in olfactory performance were found in both AD and control mice. While the visuospatial task may be more difficult than any of the odor discrimination tasks, a broad range of odor task difficulties were used to minimize this possibility.

Our finding of a lack of systematic differences in olfactory performance between the two AD mouse model strains employed here and the control mice is in contrast with several studies that reported more or less pronounced olfactory impairments in other mouse strains with AD neuropathologies. Wesson et al. [21], for example, reported an impaired response in Tg2576 mice overexpressing a mutated form of human amyloid precursor protein in an olfactory habituation/dishabituation test from 6–7 months of age on. Similarly, Macknin et al. [22] reported that Tα1-3RT mice overexpressing Tau protein displayed an impaired response in an olfactory habituation/dishabituation test at 5 months of age. Zhuo et al. [23] reported an olfactory reversal learning deficit in Tg2576 mice tested at 6 months of age, employing a digging test. This raises the question as to possible explanations underlying the discrepancy between our findings and that of the above-mentioned studies. One obvious possibility is, of course, that different strains of AD model mice may differ in the severity and/or spatial distribution of AD pathologies they express at a given age and concomitantly in the severity and type of sensory and cognitive impairment. Although the Swede mice used in the present study have been shown to express amyloid plaques at 6 months of age, a closer look at the spatial distribution and the progression of this neuropathology shows that this mouse strain expresses amyloid plaques only in the hippocampus and neocortex at this age and that the progression into other areas of the brain is much slower and possibly less severe compared to Tg2576 mice [6]. Similarly, the Tau mice used in the present study have been shown to express neurofibrillary tangles at 3 months of age, but only in the hippocampus and neocortex. Other areas of the brain such as the entorhinal cortex were only affected at 13 months of age and the cerebellum and striatum were not affected at all [4]. Thus, it may well be that differences in the severity and/or spatial and temporal distribution of AD pathologies between mouse strains might explain the differences found in the occurrence or severity of olfactory impairment between studies. This idea is also supported by Vloeberghs et al. [24] who, similar to the present findings, reported a lack of olfactory impairment in APP23 mice, another strain of AD model mice overexpressing amyloid precursor protein, when tested at 3, 6, and 12 months of age.

An alternative explanation for the discrepancy between our findings and that of the above-mentioned studies may be the behavioral test used to assess olfactory performance. Several studies that reported an AD-related impairment in olfactory performance employed a habituation/dishabituation test [e.g. 21], [22]. This type of behavioral test is not based on operant conditioning but takes advantage of an animal's spontaneous exploration of novel stimuli. Thus, it is more dependent on the motivational status of animals than operant conditioning procedures which allow for greater control of motivation. Further, the habituation/dishabituation test critically depends on the animals' overall level of motor activity and on an unimpaired motor function. Both studies mentioned above which employed this test did not report whether the overall levels of motor activity in their AD mice and control mice were comparable and whether the AD mice showed unimpaired motor function. Similarly, the digging test employed by Zhuo et al. [23] critically depends on proper motor function and on the overall level of motor activity. In contrast, the operant conditioning procedure employed in the present study uses licking at a water spout as the operant response and thus a motor behavior which is largely independent of the overall level of motor activity, is unlikely to be affected by AD pathologies, and did not differ between AD and control mice. The fact that we found marked age-related changes in olfactory performance in all three strains of mice strongly suggests that our finding of a lack of AD-related changes – both in tests based on an operant conditioning procedure (see Figs. 1 and 3) and in tests based on a habituation/dishabituation procedure (see Fig. 5) - is not due to our method not being sensitive enough to detect differences between strains. This notion is also supported by other studies which employed the same apparatus and operant conditioning method and found clear differences in olfactory performance between e.g. vasopressin 1a receptor knockout mice and controls [25], between M71 olfactory receptor monoclonal mice and controls [26], or between AMPA receptor modified mice and controls [27].

Our finding of a marked impairment in visuospatial re-learning in AD mice relative to control mice is in line with several previous studies in other strains of mice with AD pathologies. O'Leary and Brown [28], for example, reported that a double transgenic mouse strain which harbors mutant mouse/human amyloid precursor protein and presenilin-1 genes displayed visuospatial learning and memory deficits in a Barnes maze at 8–12 and 16–18 months of age. Similarly, Hsiao et al. [29] reported that a transgenic mouse strain expressing a mutant amyloid precursor protein displayed impaired visuospatial learning and memory both in a Y-maze and in a Morris water maze at 9–10 months of age, but not at 3 months of age. DeIpolyi et al. [30] found that the visuospatial impairment in their mice expressing mutant amyloid precursor protein and tested in a cross maze was present at 6 months of age, but not at 3 months of age. Boon et al. [31] showed that transgenic mice overexpressing amyloid precursor protein (APPC100.V717F mice) displayed an impairment in a visuospatial passive avoidance task at 4–9 and 16–22 months of age. In these studies the hippocampus, which is known to be critically involved in visuospatial learning and memory, was affected by AD pathologies at the age when the impairment occurred. The same is true for the two AD mouse strains tested in the present study [4], [6]. The fact that visuospatial deficits occurred consistently in different strains of AD mice and using different tests of visuospatial capabilities suggests that this non-olfactory impairment may be a more robust phenotypical trait of AD pathologies in mice than olfactory impairment, though task difficulty may be an important factor.

**Figure 6:**
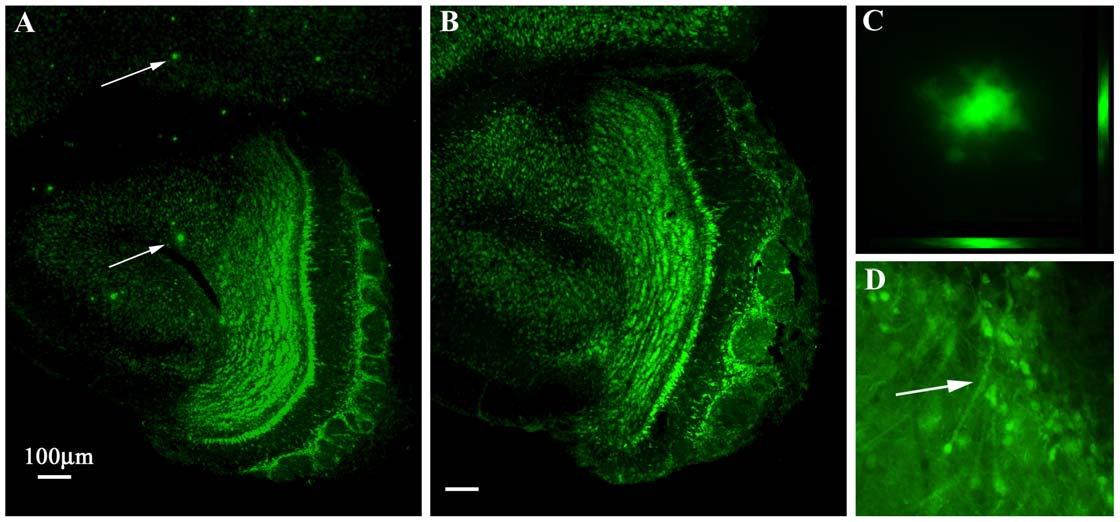
Histology. After fixation of 18 month-old animals, 50 µm coronal sections were stained with Thioflavin T to label β-amyloid plaques. A) Swede mouse. White arrows indicate plaques in the orbital frontal cortex and anterior olfactory cortex, respectively. B) 18 month-old control mouse without any plaque staining. C) A Z-stack showing a 10 µm diameter plaque, one of the larger ones observed. D) A middle tufted cell of the olfactory bulb showing Tau tangle phenotype in a tested Tau mouse.

Our finding of a marked age-related decrease in olfactory performance both in the AD-mice and in the control mice is in agreement with the well-documented decrease in human olfactory capabilities with age [e.g. 32], [33]. This finding suggests that the lack of AD-related changes in olfactory performance that we found is unlikely to be an artifact or the result of an insensitive method. Rather, it is in line with the notion that the mechanisms underlying AD-related and age-related changes in olfactory performance may differ from each other. This notion is supported by recent studies which found that an age-related olfactory decline in humans may be associated with changes in the expression of brain-derived neurotrophic factor and thus with a mechanism thought to be independent of the etiology of AD [34]. Our finding of a complete lack of β-amyloid plaques and Tau-associated neurofibrillary tangles in the 18-month old control mice lends additional support to the notion that the histologically- confirmed presence of this AD-related neuropathology in the AD mouse model strains used here can not explain the observed age-related olfactory impairment (Fig. 6).

Whereas numerous studies assessed histological and physiological changes in the aging olfactory system in mice [e.g. 35]–[37], surprisingly few studies so far assessed age-related changes in olfactory performance in mice at the behavioral level. Enwere et al. [38] reported that 24-month old C57BL/6 mice displayed impaired olfactory discrimination capabilities compared to 2-month old animals. Using the same mouse strain, Patel and Larson [39] found that 24-month old animals had a lower olfactory sensitivity and needed more trials to criterion in odor discrimination tasks than 4-month old mice. Unfortunately, neither study tested age classes in between 4 months and 24 months of age. Nevertheless, these results are consistent with our finding that olfactory sensitivity, discrimination learning and long-term odor memory markedly decreased between 15 and 18 months of age in both our AD and control mice (see Fig. 3).

Taken together, the results of the present pilot study suggest that transgenic mouse strains expressing the same type of neuropathologies as are typical of human AD do not necessarily display the same types of sensory or cognitive impairments as humans typically do. Thus, each AD model mouse strain should be carefully assessed for phenotypical traits when intended to serve as a valid and useful model of human AD. While the number of animals tested for this study was low, their performance in olfactory discrimination and visuospatial learning tasks was robust and significant. Future studies should focus on developing a robust odor identification assay for mouse models to explore the behavioral and pathological correlates between AD model mice and human AD patients.
